# Effects of soft rock on soil properties and bacterial community in Mu Us Sandy Land, China

**DOI:** 10.7717/peerj.13561

**Published:** 2022-06-21

**Authors:** Zhen Guo, Wei Hui, Juan Li, Chenxi Yang, Haiou Zhang, Huanyuan Wang

**Affiliations:** 1 Shaanxi Provincial Land Engineering Construction Group Co., Ltd., Xi’an, Shaanxi, China; 2 Institute of Land Engineering and Technology, Shaanxi Provincial Land Engineering Construction Group Co., Ltd., Xi’an, Shaanxi, China

**Keywords:** Soil properties, Community structure, Diversity, High-throughput sequencing, Mu Us Sandy Land

## Abstract

Soft rock is a new material that could be used for the improvement of Mu Us Sandy Land, China. It can be utilized for wind prevention and sand fixation, both of which are of great importance to ecological restoration aims and cultivated land replenishment in desert areas. Four treatments with different compound ratios of soft rock and sand—0:1 (CK), 1:5 (P1), 1:2 (P2), and 1:1 (P3)—were studied. Fluorescence quantitative PCR (qPCR) and high-throughput sequencing were used to analyze the structure and diversity of the bacterial community in the compound soil and its relationship with physical and chemical parameters in the soil. The results showed that in comparison to CK treatment, soil organic carbon (SOC), total nitrogen (TN), and NH_4_^+^-N increased significantly in the P1 treatment; available phosphorus (AP), available potassium (AK), and NO_3_^−^-N increased significantly in the P3 treatment. The bacterial gene copy number with P3 treatment was highest, showing a significant increase of 182.05% compared with the CK treatment. The three bacterial groups with the highest relative abundance at the phylum level were *Actinobacteria*, *Proteobacteria*, and *Chloroflexi*, accounting for more than 70% of the total population. The bacterial α diversity showed the same trend, the diversity and abundance indices of the P1 and P3 treatments were the highest, and the β diversity showed that the community structure of the two groups in these treatments were similar. *norank_f__Roseiflexaceae* and *Gaiella* (belonging to *Actinobacteria*) significantly differed with differing compound ratios in each treatment. NO_3_^−^-N, NH_4_^+^-N and SOC were the main factors affecting bacterial community structure, and had a significant positive correlation with *Gaiella* abundance. These species are known to play an important role in stabilizing the soil structure of sandy land. Overall, 1:5 and 1:1 compound soil mixtures were beneficial towards the microbial community of sandy land, which plays an important role in biological sand fixation. This study provides an important theoretical basis for the supplementation of arable land area and the improvement of sandy land productivity, and has a good promotion prospect.

## Introduction

Soil bacteria are one of the most diverse, abundant, and functional groups of soil organisms (Steenwerth et al., 2003). They are important drivers of biogeochemical cycles, participate in the transformation of soil nutrients, and are key in ecosystem matter cycles and energy flow (Nacke et al., 2011). The structure and function of the soil microbial community is an important indicator reflecting soil quality and fertility (Chen et al., 2020). With increasing human disturbances to soil such as changes in land regulation, fertilization methods, and planting regimes, many studies have shown that these disturbances have a significant impact on the structure, diversity, and function of the soil microbial community (Steenwerth et al., 2003). The dominant factors driving microbial community structure changes vary across types of soil; especially in the newly improved soil found in Mu Us Sandy Land, China, the trends of microbial community change still need to be further explored.

The Mu Us Sandy Land is located in a semi-arid region of northern China, a compound ecosystem area composed of grassland, forestry, and agriculture regions (Zhang et al., 2020). The area is susceptible to human activities and serious soil wind erosion (Li et al., 2017). Mu Us Sandy Land is rich in light and heat resources, and land consolidation in this area could help guarantee the quantity of cultivated land and food security in China (Han, Xie & Zhang, 2012; Guo, Zhang & Wang, 2021). A large number of scholars have studied the impact of agricultural use patterns on the soil quality of farmland in sandy land in different experimental areas; results have indicated that reasonable land-use methods and appropriate farming management measures can lead to increased soil carbon and nitrogen storage in the regional habitat (Di et al., 2016). Conversely, over-use of land can reduce soil quality, leading to a decline in land productivity (Zhu et al., 2020). Liu & Zhao (2010) found that conservation tillage and fine management of irrigated farmland were beneficial to soil improvement and ecosystem restoration in sandy land areas. Su et al. (2017) showed that after an area of desert sandy land was reclaimed into farmland, though the soil fertility significantly improved over time, the soil fertility in the area was still low. He et al. (2020) used an engineering measure to improve sandy land, indicating that soft rock (a loose rock widely distributed in the Mu Us Sandy Land) mixed with aeolian sandy soil significantly improved the water and fertilizer retention capacity of the sandy land studied. As soft rock is believed to become soft as mud when exposed to water, it could also improve the chemical and physical characteristics of sandy soil, increasing crop productivity along with the colloidal content of the soil (Guo, Zhang & Wang, 2021). Using soft rock to improve aeolian sandy soil in the Mu Us Sandy Land could improve water and fertilizer retention capacity, increase the cultivatable land area, and increase crop yields, improving the area and maintaining the sustainable development of the regional ecological environment (Zhang et al., 2020; Han, Xie & Zhang, 2012).

Liu et al. (2019) believed that bacterial community compositions in desert areas have been greatly disturbed from their original environmental conditions. Du et al. (2017) found that the abundance and diversity of microorganisms are the highest on the surface. The improvement of sandy land by soft rock is an engineering measure to restore sandy land, and study of the influence of soft rock on soil bacteria in sandy land is important for revealing the response mechanism of the underground microbial community. Previous studies on compound soil made of soft rock and sand mainly focused on physical structure and chemical properties; there are few reports on the differences in soil bacterial community structure and factors driving this change during soil development. This study examined compound soils made of different proportions of soft rock and sand tested in the Mu Us Sandy Land, and used high-throughput sequencing technology to analyze the bacterial community structure and diversity of the 0–20 cm soil (Xiang et al., 2021). The aims were to clarify the differences in the bacterial community structure in the compound soil, reveal the synergistic effect of soil environmental factors with the bacterial community, and provide a theoretical basis for sandy land and soil fertility improvement.

## Materials and Methods

### Overview of the test site

The test field was located in the Mu Us Sandy Land (E109°28′58″–109°30′10″, N38°27′53″–38°28′23″) in Yuyang District, Yulin City, northwest Shaanxi, China; the altitude was between 1,206–1,215 m. A long-term positioning observation test station in the field was used. The test area belongs to a typical mid-temperate semi-arid continental monsoon climate zone, with uneven temporal and physical distribution of precipitation, a dry climate, a long winter, a short summer, four distinct seasons, and sufficient sunshine. The average annual temperature is 8.1 °C, the average annual frost-free period is 154 days; the average annual precipitation is 413.9 mm, and 60.9% of the rainfall is concentrated over the three months from July to September. The annual average sunshine hours are 2,879, and the sunshine percentage is 65%. The soil type in the project area is mainly sandy soil.

### Experiment design

The test field was used to simulate the mixed layer of soft rock and sand in the Mu Us Sandy Land. The experimental plot had a mixture of soft rock and sand laid at 0–30 cm depth. The selected ratio of soft rock to sand for each treatment (0:1, CK; 1:5, P1; 1:2, P2; 1:1, P3) was repeated three times with an area of 5 m × 12 m (60 m^2^). This volume ratio was chosen because of the area suitable for crop planting, and the soil structure in this area had good ventilation and water permeability. The field trial implemented a potato cropping system once a year, planted in mid-April and harvested in mid-to-late September; an artificial planting mode was adopted throughout the year. The test fertilizer types were urea, diammonium phosphate, and potassium chloride. The fertilizer application amount was N = 300 kg ha^−1^, P_2_O_5_ = 375 kg ha^−1^, and K_2_O = 180 kg ha^−1^ per year. All phosphate fertilizers and potash fertilizers were used as base fertilizers, and 50% of nitrogen fertilizers were used as base fertilizers. 1 to 2 days before planting, the three kinds of fertilizers were weighed out according to the required amount for each plot, were mixed evenly, and sprinkled evenly on the soil surface before properly raking to mix with the topsoil. The remaining 50% of nitrogen fertilizer was applied 20% in the potato seedling stage and 30% after flowering. Irrigation of the experimental field used a drip irrigation mode. Irrigation in the early stage (from sowing to the beginning of tuber formation) kept the relative soil moisture content at 60–70%. The relative water content of the field soil was maintained at no less than 60% during the mid-term irrigation (from the beginning of tuber formation to the fall of flowers), and the overall water content was maintained at about 65%. In the later period of irrigation, the amount of irrigation was controlled below 10 mm to prevent white spots on the skin of the potatoes.

### Soil sample collection

After the potato harvest in September 2021, soil samples of the 0–20 cm soil layer were collected from each plot. Three mixed soil samples were collected from each plot, and each sample was uniformly mixed by the five-point method. The collected soil samples were divided into two parts after removing animal and plant residues; one was naturally air-dried and filtered through 1 and 0.149 mm screens for use in determining soil physical and chemical properties, and the other was stored in a refrigerator at −80 °C for microbial analysis (Tchakounté et al., 2018).

### Determination of soil physical and chemical properties

Soil organic carbon (SOC) was determined by the potassium dichromate-concentrated sulfuric acid external heating method (Nelson & Sommer, 1996; Mann et al., 2019). Total nitrogen (TN) was determined by Kjeldahl digestion. Available phosphorus (AP) was determined by molybdate blue colorimetry, and available potassium (AK) concentrations were measured using atomic absorption spectrometry (Dai, Wang & Fu, 2017; Chun et al., 2021). NO_3_^−^-N and NH_4_^+^-N were extracted at a ratio of 10 g fresh soil to 100 mL 2 M KCl. After shaking for 1 h, the extracts were filtered and analyzed by a continuous flow analytical system (San++ System, Skalar, Holland) for NO_3_^−^-N and NH_4_^+^-N (Di et al., 2016). pH was measured using a pH meter (PHS-3E: INESA, Shanghai, China) with the soil-to-water ratio at 1:5 (Minasny et al., 2011).

### Soil DNA extraction and sequencing

The E.Z.N.A. ®Soil DNA Kit (Omega, Inc., Norwalk, CT, USA) was used to extract total DNA from the soil sample; DNA concentration and purity were determined by NanoDrop 2,000 spectrophotometer and visually analyzed by 1% agarose gel electrophoresis (Luo et al., 2019). Using the total microbial DNA of each soil sample as a template, PCR amplification was carried out with 16S rRNA V3-V4 region primers 338F (5′-ACTCCTACGGGAGGCAGCAG-3′) and 806R (5′-GGACTACHVGGGTWTCTAAT-3′) (Ma et al., 2020). The PCR products were recovered and purified by 2% agarose gel, eluted in Tris-HCl, and detected by 2% agarose electrophoresis. QuantiFluorTM-ST (Promega, Madison, WI, USA) was used for quantitative detection. According to the standard operating procedures of the Illumina MiSeq platform (Illumina, SanDiego, CA, USA), the purified amplified fragments were constructed into a PE 2 × 300 library. Sequencing was performed using Illumina’s MiSeq PE300 platform (Soliman et al., 2017; Chen et al., 2020).

### Fluorescent qPCR amplification

Fluorescent qPCR was performed using the same primers as the high-throughput sequencing detailed above. A reaction mix of 20 μL consisted of 10 μL 2X ChamQ SYBR Color qPCR Master Mix, 0.8 μL each of upstream and downstream primer (5 μmol L^−1^), 2 μL template, 0.4 μL 50X ROX Reference Dye 1, and 6 μL ddH_2_O. The amplification program was 95 °C pre-denaturation for 3 min; 95 °C denaturation for 5 s, 58 °C annealing for 30 s, and 72 °C extension for 1 min (Hassan et al., 2021). An ABI7300 fluorescence quantitative PCR instrument (Applied Biosystems, Waltham, MA, USA) was used for amplification and measurement. Three replicates were used for each sample the final gene abundance was calculated based on the soil dry weight.

### Data processing and analysis

The experimental data was analyzed for variance with SPSS 20.0. QIIME (Version 1.9.1) was used to analyze the composition of the sample and obtain the bacterial community composition and relative abundance at different taxonomic levels. The relative abundance maps at the phylum and genus levels were compared with the corresponding microbial databases (silva138/16s_bacteria) (Gosai et al., 2018). QIIME (Version 2.0) was used to analyze the dilution curve based on OTU and calculate the species diversity index. R (Version 3.3.1) was used to draw the principal component analysis (PCA) diagram of the soil bacterial community structure. Canoco was used to perform a redundancy analysis (RDA) between bacterial community composition and environmental factors. The Spearman correlation coefficient was used to analyze the correlation between environmental factors and species, and the heat map was drawn with the aid of R.

## Results

### Soil properties

The SOC content in the P1 and P2 treatments was higher by 46.07% (*n* = 12, df = 3, *P* = 0.0253) and 43.46% (*n* = 12, df = 3, *P* = 0.0284), respectively, compared with the CK treatment. The TN content of the P1 treatment was significantly increased by 112.50% compared to CK, though there was no significant difference between the P1, P2, and P3 treatments. The changing trend of NH_4_^+^-N content was consistent with changes seen for TN. The contents of AP, AK, and NO_3_^−^-N were highest in P3, and the NO_3_^−^-N content increased with the increase of soft rock content. There was no significant difference in pH between treatments. The abundance of 16S rRNA genes in the four mixed soil treatments was between 0.39 × 10^9^–1.10 × 10^9^ copies g^−1^ dry soil; the P3 treatment had the highest bacterial gene copy number, 17.02%, 155.81%, and 182.05% greater than in P1, P2, and CK treatments, respectively. There was no significant difference between P2 and CK gene copies (Table 1).

**Table 1:** Soil properties under different compound ratio treatments.

Treatments	SOC (g kg^−1^)	TN (g kg^−1^)	AP (mg kg^−1^)	AK (mg kg^−1^)	NO_3_^−^-N (mg kg^−1^)	NH_4_^+^-N (mg kg^−1^)	pH	Genes copies number (×10^9^)
CK	1.91 ± 0.47 b	0.16 ± 0.04 b	3.85 ± 0.87 c	38.52 ± 4.39 c	4.23 ± 0.33 d	2.39 ± 0.42 b	8.89 ± 2.11 a	0.39 ± 0.03 c
P1	2.79 ± 0.21 a	0.34 ± 0.06 a	7.57 ±2.14 b	63.47 ± 6.38 b	13.08 ±1.96 c	3.77 ± 1.43 a	8.78 ± 0.91 a	0.94 ± 0.05 b
P2	2.74 ± 0.15 a	0.24 ± 0.05 ab	5.53 ± 1.52 c	44.26 ± 4.56 c	17.44 ± 4.05 b	3.44 ± 0.88 a	8.87 ± 1.74 a	0.43 ± 0.06 c
P3	2.58 ± 0.09 ab	0.27 ± 0.07 ab	14.84 ± 3.55 a	72.11 ± 5.61 a	18.64 ± 2.21 a	3.45 ± 1.02 a	8.56 ± 1.22 a	1.10 ± 0.14 a

**Note:**

CK, the volume ratio of soft rock to sand is 0:1; P1, the volume ratio of soft rock to sand is 1:5; P2, the volume ratio of soft rock to sand is 1:2; P3, the volume ratio of soft rock to sand is 1:1. SOC stands for soil organic carbon; TN stands for soil total nitrogen; AP stands for available phosphorus; AK stands for available potassium; NO_3_^-^-N stands for nitrate nitrogen; NH_4_^+^-N stands for ammo-nium nitrogen. Lowercase letters indicate significant differences at the 5% level between different treatment.

### Bacterial community composition

**Figure 1:**
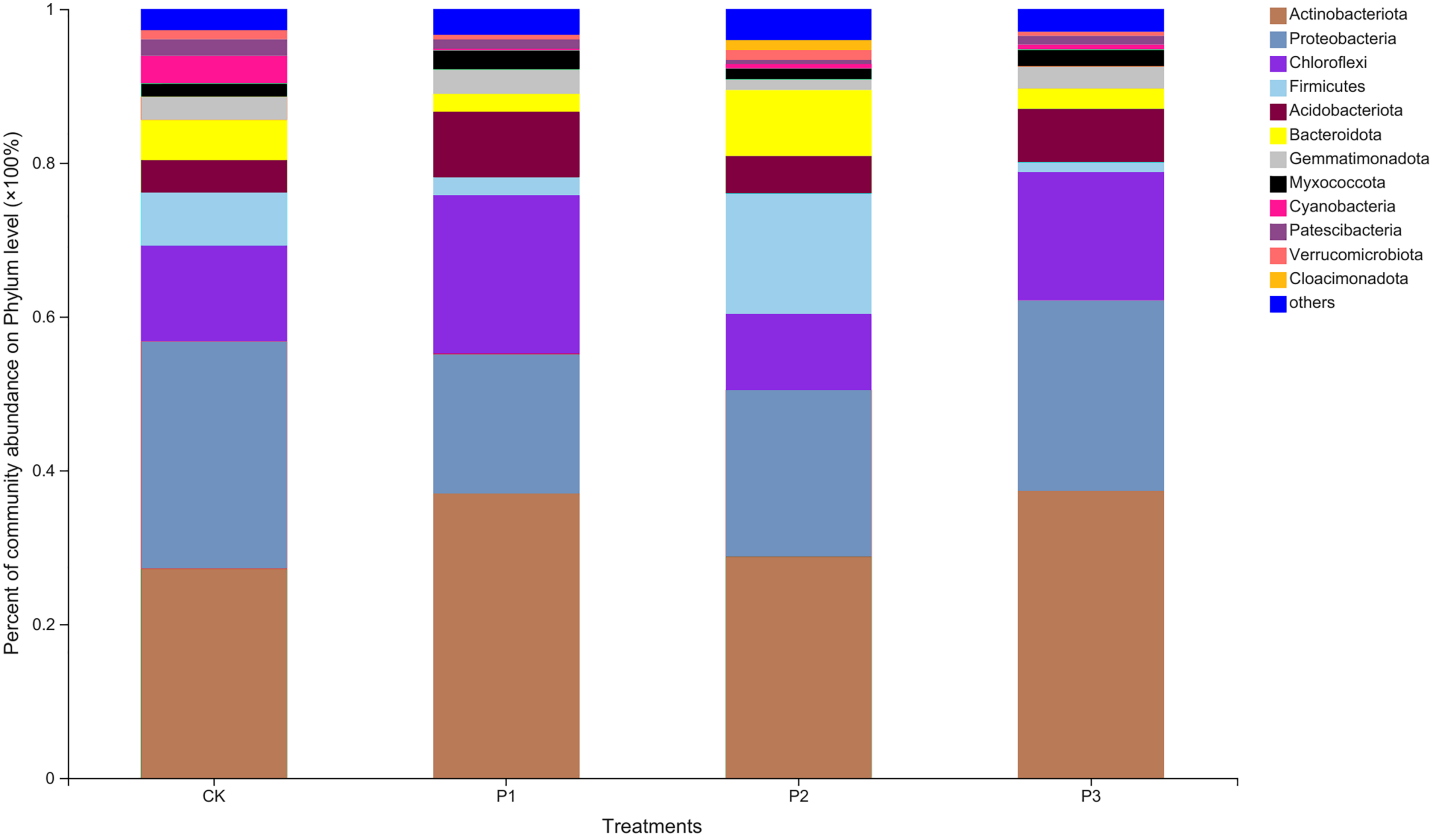
Based on phylum level bacterial community composition. CK, the volume ratio of soft rock to sand is 0:1; P1, the volume ratio of soft rock to sand is 1:5; P2, the volume ratio of soft rock to sand is 1:2; P3, the volume ratio of soft rock to sand is 1:1.

Twelve abundant bacterial phyla were found in the soil samples; others with relative abundances less than 0.01 were grouped into one category (Fig. 1). The three most dominant phyla, in order of abundance, were *Actinobacteriota*, *Proteobacteria*, and *Chloroflexi*. *Cloacimonadota*, a unique bacterial phylum, appeared only in the P2 treatment. Compared with the CK treatment, the relative abundance of *Actinobacteriota* in the P3 treatment increased by 37.82%. *Proteobacteria* in other treatments all showed a decreasing trend compared to CK, with a larger decrease in abundance observed in P1. Compared with CK, *Chloroflexi* abundance decreased by 20.56% under P2 treatment, but showed an increase in P1 and P3 treatments.

**Figure 2:**
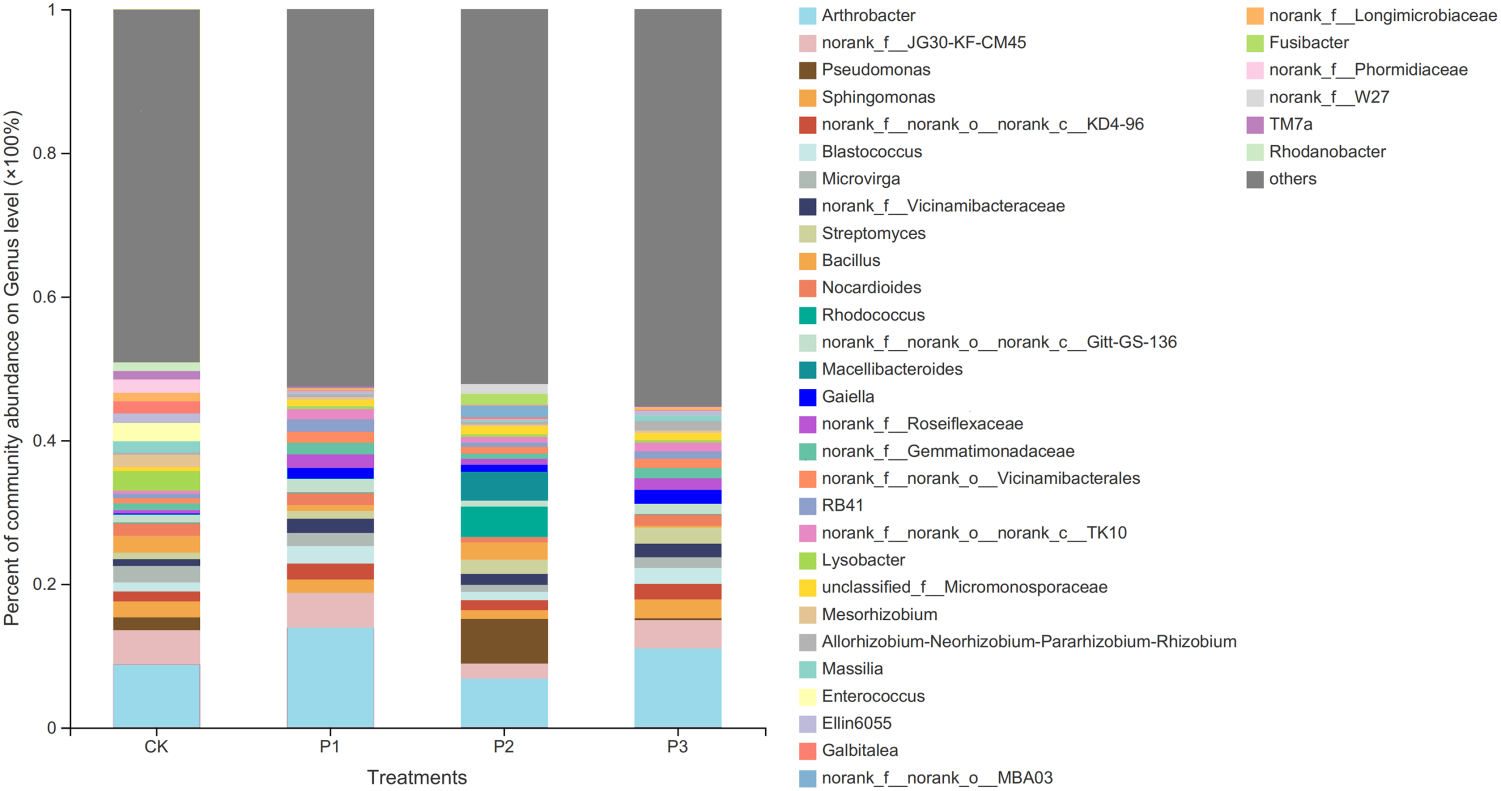
Based on genus level bacterial community composition. CK, the volume ratio of soft rock to sand is 0:1; P1, the volume ratio of soft rock to sand is 1:5; P2, the volume ratio of soft rock to sand is 1:2; P3, the volume ratio of soft rock to sand is 1:1.

At the genus level, the differences between bacteria in the different treatments increased (Fig. 2). The dominant bacteria in CK were *Arthrobacter* (6.33%), *norank_f__JG30-KF-CM45* (5.48%), and *Lysobacter* (4.54%). The dominant bacteria in P1 were *Arthrobacter* (13.90%), *norank_f__JG30-KF-CM45* (4.83%), and *Blastococcus* (2.42%). The dominant bacteria in P2 were *Arthrobacter* (6.79%), *Pseudomonas* (6.23%), and *Rhodococcus* (4.26%). The dominant bacteria in P3 were *Arthrobacter* (11.01%), *norank_f__JG30-KF-CM45* (3.93%), and *Sphingomonas* (2.64%).

### Bacterial α diversity

The coverage values in this study were all greater than 97%, indicating that the sequencing results were highly reliable for all samples and covered most of the community present (Table 2). A total of 216,708 sequences were obtained (P2 > P1 > P3 > CK); the number of OTUs decreased from P1 to P2 to P3 to CK (Table 2). Chao and Ace indexes represent the abundance of bacterial communities, and the higher the value, the higher the abundance of a species. The Chao indices of the P1 and P3 treatments were significantly higher than that of the P2 and CK treatments. Trends in the Ace index were consistent with that of the Chao index. The Shannon index represents the diversity of the bacterial community; the larger the Shannon index, the higher the diversity. The results showed that the addition of soft rock promoted the increase of bacterial diversity in sandy soil, but there was no significant difference between different treatments.

**Table 2:** Bacterial diversity of under different compound ratio treatments.

Treatments	Reads	OTUs	Chao	Ace	Shannon	Coverage (%)
CK	46,213	3,125	2,947.17 ± 50.81 b	2,916.35 ± 49.85 b	5.67 ± 0.60 a	98.07
P1	55,139	3,909	4,274.92 ± 40.56 a	4,233.60 ± 42.11 a	6.23 ± 1.02 a	97.04
P2	60,610	3,776	2,789.11 ± 90.22 b	2,821.63 ± 88.21 b	5.89 ± 0.98 a	98.40
P3	54,746	3,770	3,937.10 ± 83.41 a	3,907.27 ± 90.42 a	6.19 ± 0.99 a	97.39

**Note:**

CK, the volume ratio of soft rock to sand is 0:1; P1, the volume ratio of soft rock to sand is 1:5; P2, the volume ratio of soft rock to sand is 1:2; P3, the volume ratio of soft rock to sand is 1:1. Lowercase letters indicate significant differences between different compound ratios (*P* < 0.05).

### Bacterial community β diversity

**Figure 3:**
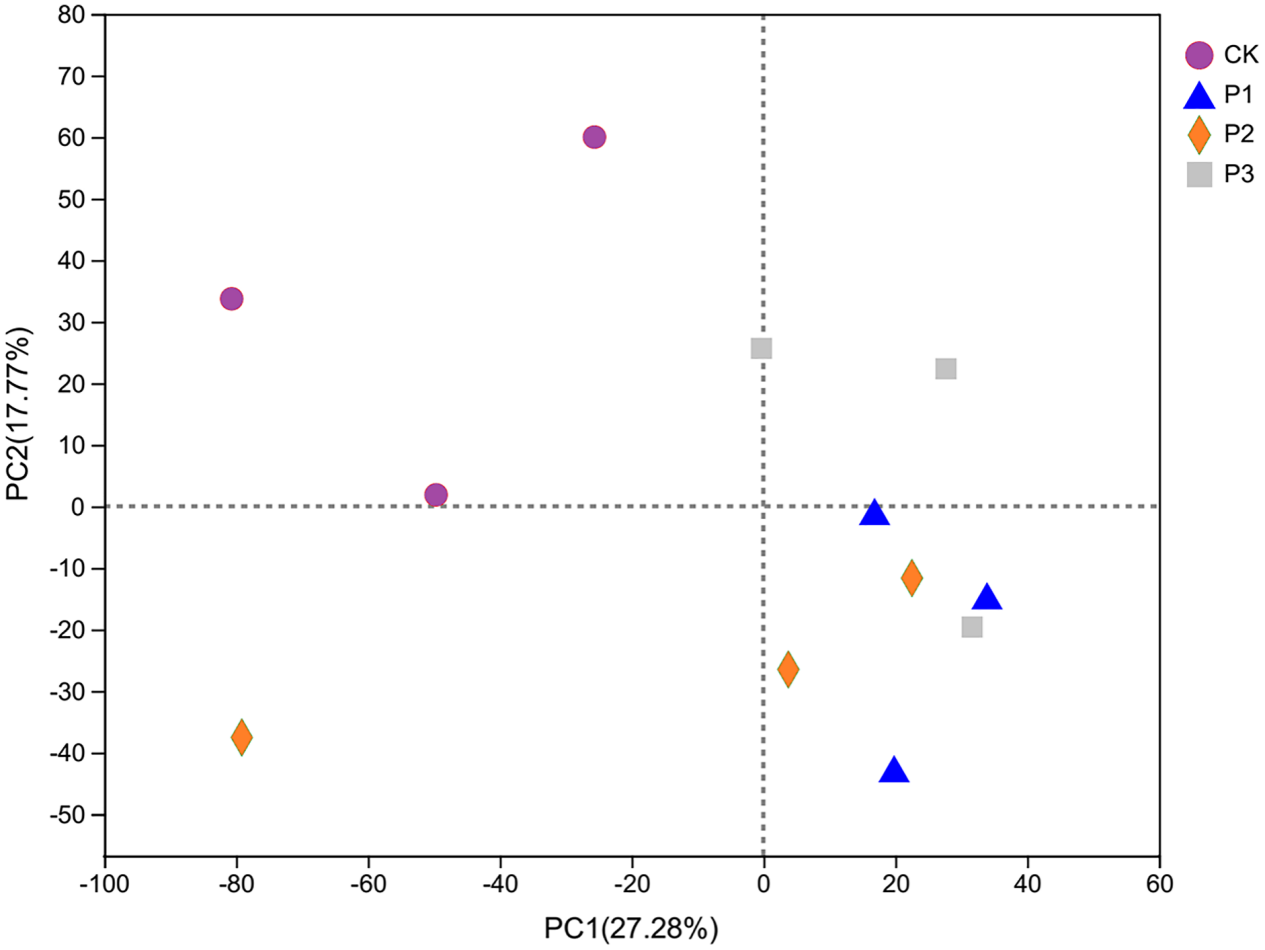
The PCA analysis of bacterial community based on OTU level. CK, the volume ratio of soft rock to sand is 0:1; P1, the volume ratio of soft rock to sand is 1:5; P2, the volume ratio of soft rock to sand is 1:2; P3, the volume ratio of soft rock to sand is 1:1.

PCA analysis showed that CK was clearly different from other processed samples on the PC1 axis, as other samples were located to the right of CK (Fig. 3). The results therefore showed that the addition of soft rock had an impact on the bacterial community structure of sandy soil. The PC1 and PC2 axes explained 27.28% and 17.77% of the total variation, respectively. The distance between the P1 and P3 soil samples was relatively small, indicating that their bacterial community compositions were similar. The P2 treatment had less overlap with P1 and P3, and therefore this treatment’s microbial community had lower similarity with the others.

### Analysis of species differences between treatments

**Figure 4:**
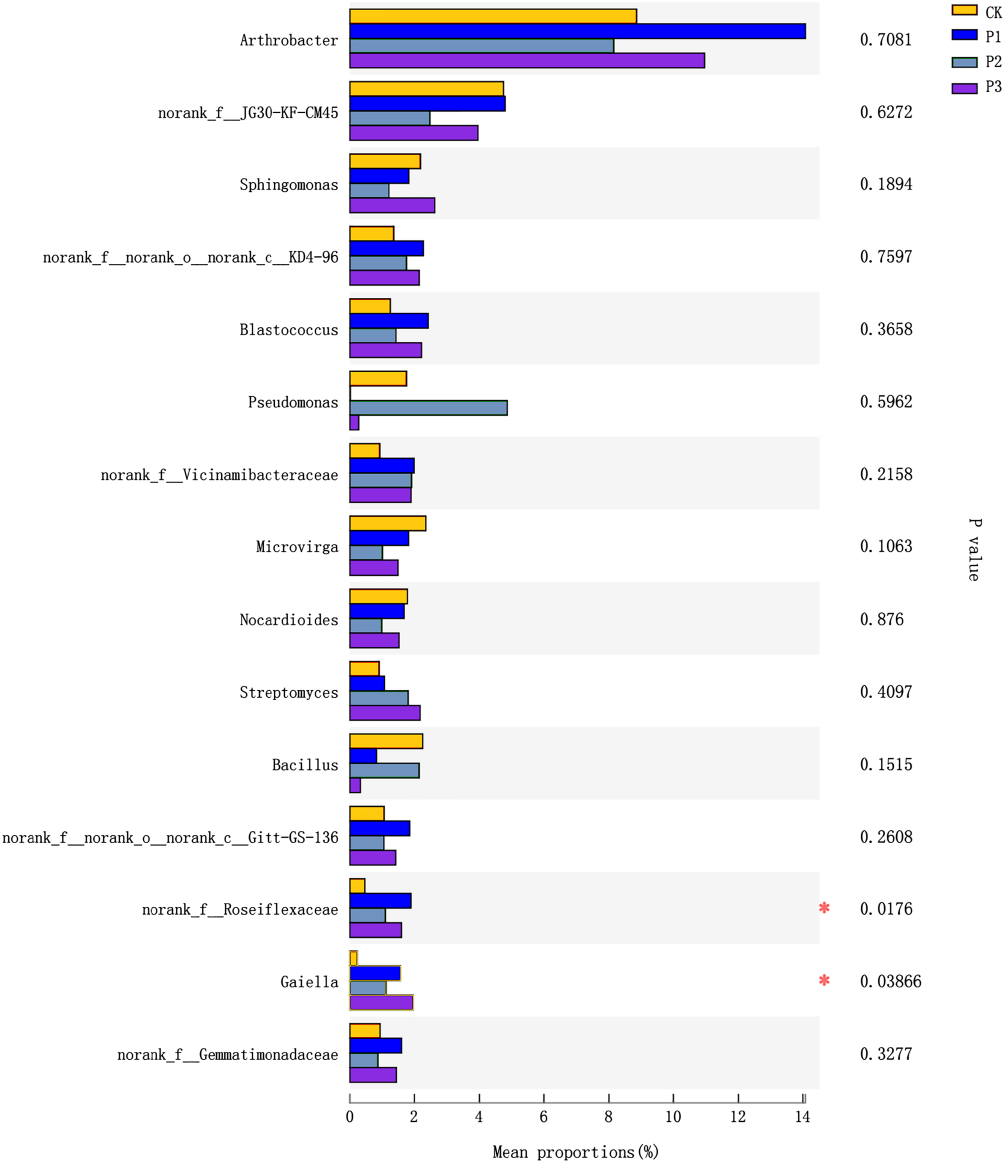
Differences between treatments of bacteria at the Genus level. CK, the volume ratio of soft rock to sand is 0:1; P1, the volume ratio of soft rock to sand is 1:5; P2, the volume ratio of soft rock to sand is 1:2; P3, the volume ratio of soft rock to sand is 1:1. An asterisk (*) indicates significant differences at the 5% level between different treatment.

The top 15 species in terms of relative abundance at the genus level were selected for use in analyzing differences between treatments. *norank_f__Roseiflexaceae* and *Gaiella* (belonging to the *Actinobacteria* phylum) showed significant differences between different treatments (Fig. 4). The *norank_f__Roseiflexaceae* was at highest abundance in the P1 treatment, followed by the P3 and P2 treatments; the CK treatment had the lowest abundance. Compared with the CK treatment, the relative abundance of *Gaiella* increased significantly in the treated soils, with P3 being the highest, followed by P1, and lastly P2.

### The relationship between soil properties and bacterial communities

**Figure 5:**
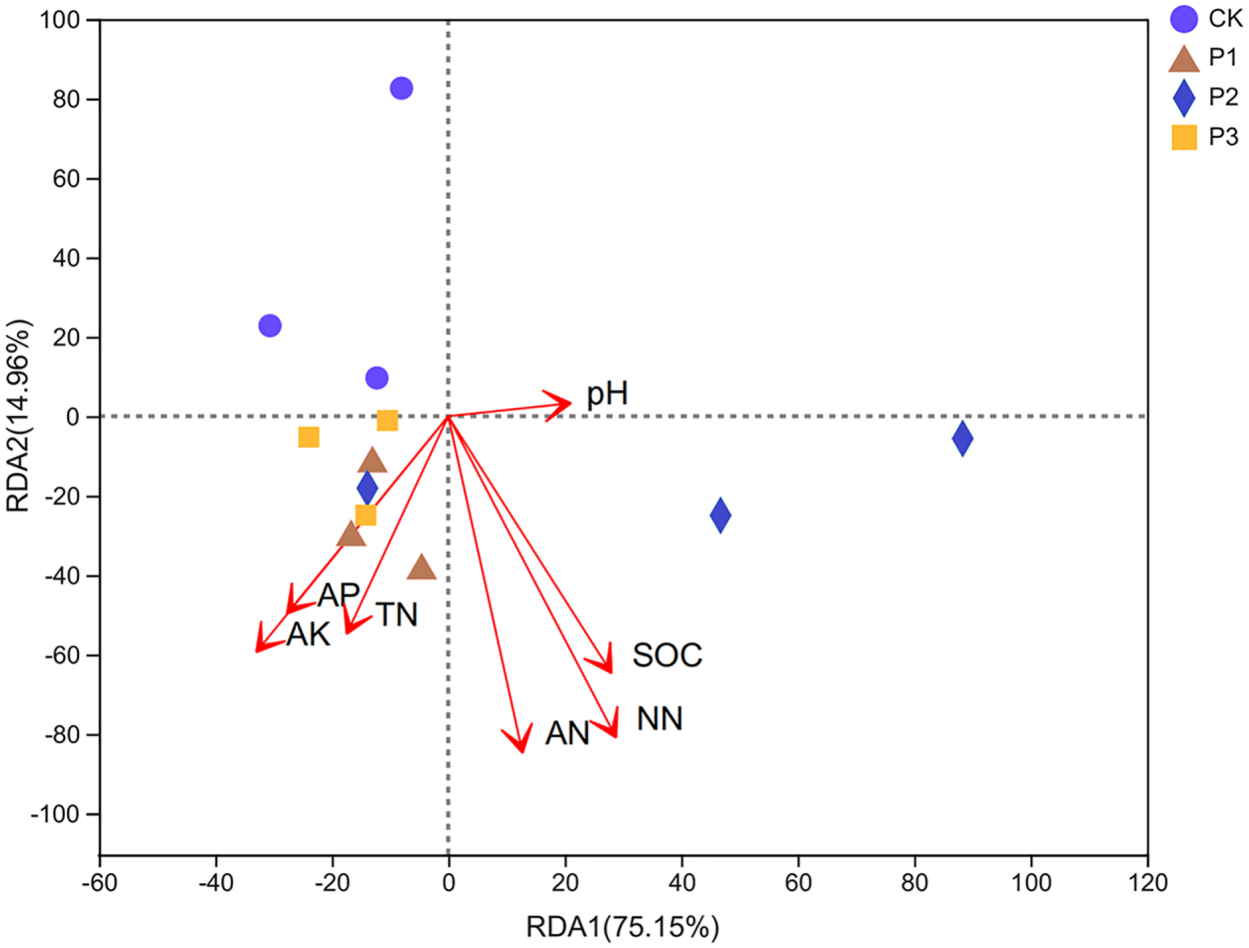
Redundancy analysis (RDA) of bacterial community composition and soil chemical properties. SOC stands for soil organic carbon; TN stands for soil total nitrogen; AP stands for available phosphorus; AK stands for available potassium; NN stands for NO_3_^−^-N; AN stands for NH_4_^+^-N.

In the 0–20 cm soil layer, the interpretation degrees of the RDA1 axis and RDA2 axis were 75.15% and 14.96%, respectively, and the sum of the two axes was 90.11%. The degree of influence of various environmental factors on the composition of bacterial communities in soil samples was NN (NO_3_^−^-N), AN (NH_4_^+^-N), and SOC with the greatest impact; AK, AP, and TN had the second highest impact, and pH had the least impact. The bacterial community compositions of P1 and P3 were positively correlated with other factors aside from pH (Fig. 5). The bacterial community composition of P2 was positively correlated with factors other than AP, AK, and TN.

### Heat map of the correlation between soil properties and bacterial communities

**Figure 6:**
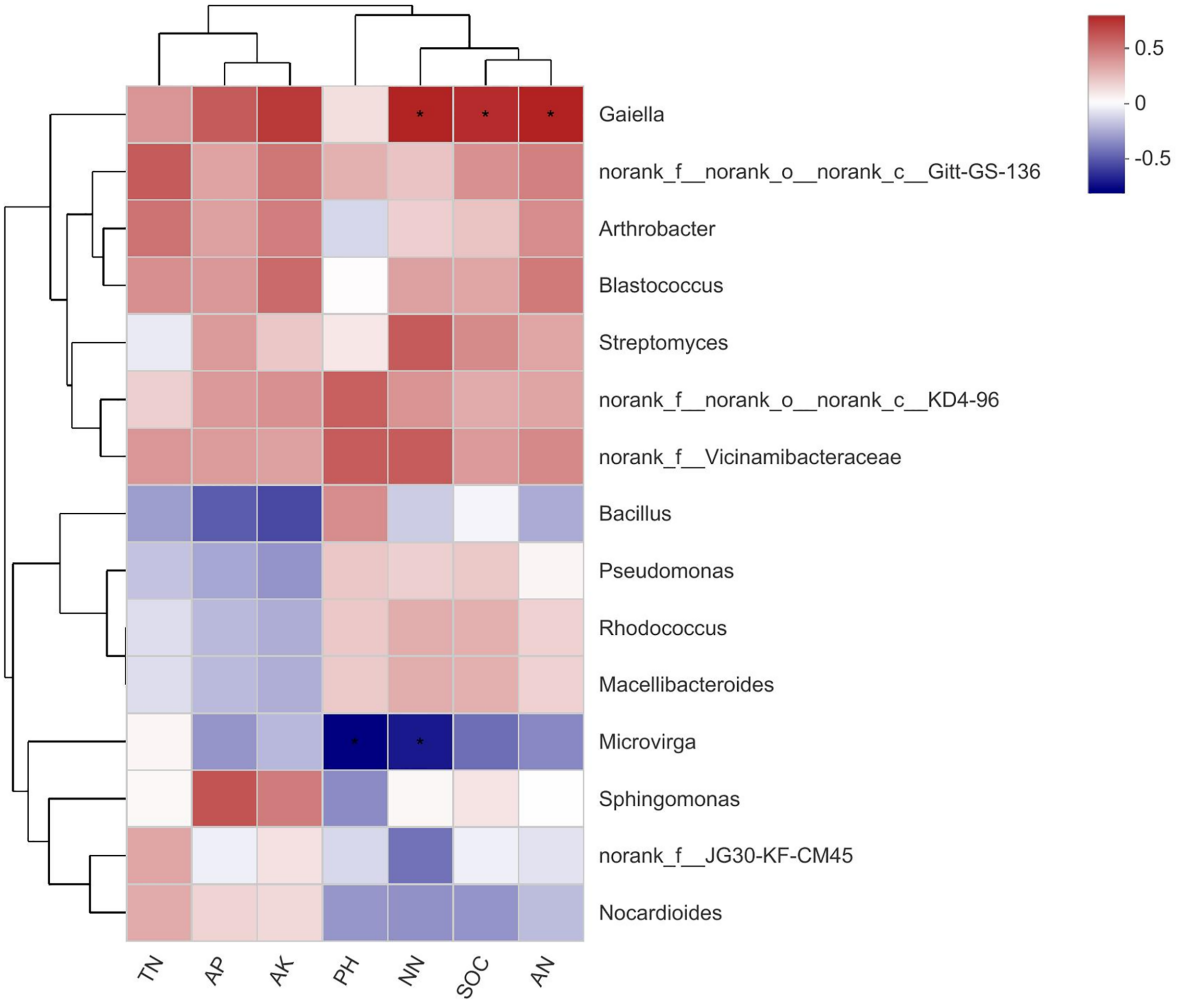
A correlation heatmap of soil bacteria and soil chemical properties at the Genus level in different soil layers. SOC stands for soil organic carbon; TN stands for soil total nitrogen; AP stands for available phosphorus; AK stands for available potassium; NN stands for NO_3_^−^-N; AN stands for NH_4_^+^-N. If the *P* value is less than 0.05, it is marked with an asterisk (*).

The top 15 abundant species at the genus level were selected for correlation analysis with soil properties. The results showed that *Gaiella* (Belonging to the *Actinobacteria* phylum) was significantly correlated with NN, SOC, and AN, while *Microvirga* (belonging to the *Proteobacteria* phylum) was significantly correlated with pH and NN (Fig. 6). There were no significant differences between other bacterial genera and soil properties.

### Bacteria functional differences of compound soil

**Figure 7:**
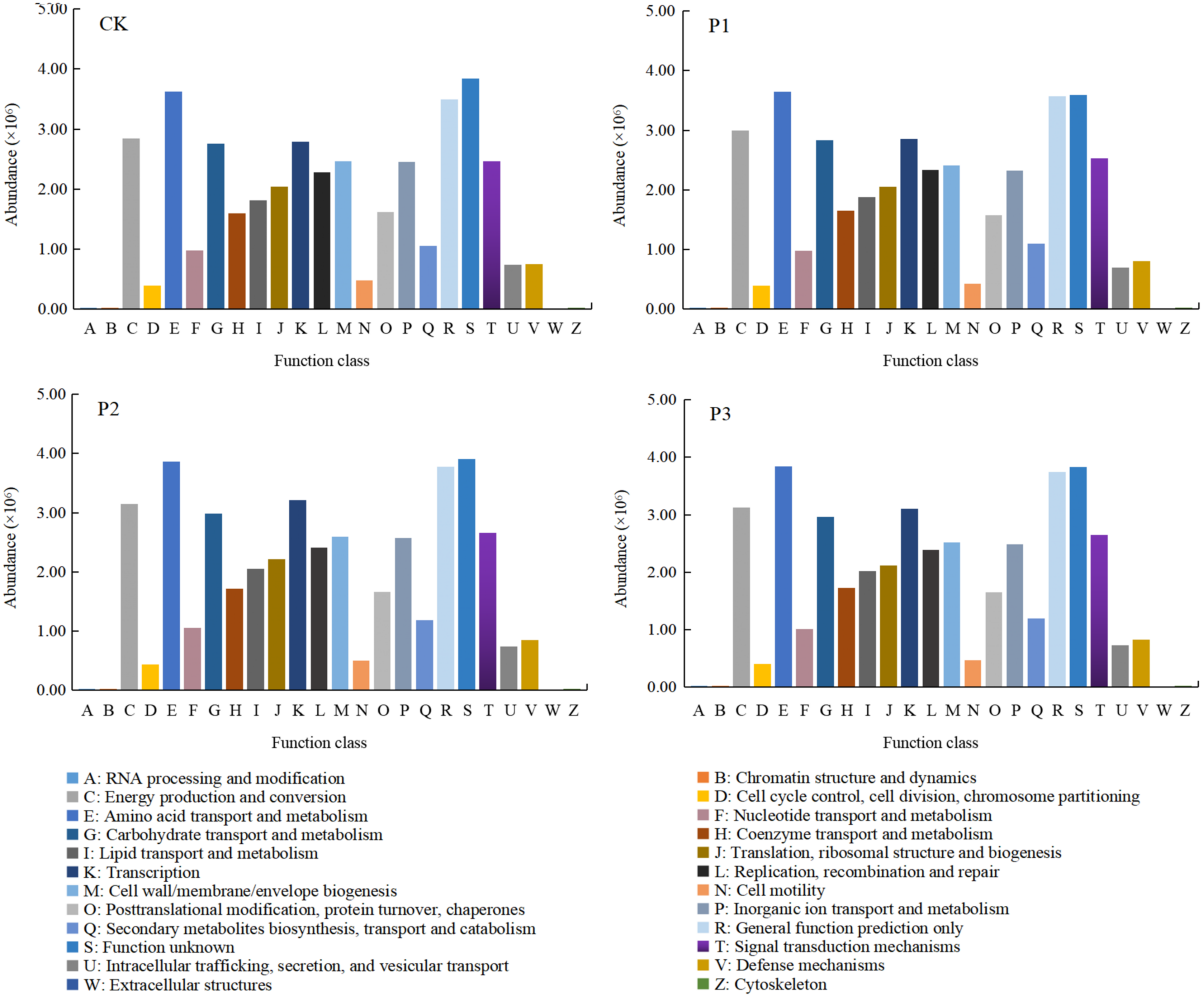
The functional differences of bacterial communities in different composite treatments of soft rock and sand. CK, the volume ratio of soft rock to sand is 0:1; P1, the volume ratio of soft rock to sand is 1:5; P2, the volume ratio of soft rock to sand is 1:2; P3, the volume ratio of soft rock to sand is 1:1.

After soft rock improved the sandy land, the function of soil bacteria showed obvious differences (Fig. 7). The abundances of C (energy production and conversion), E (amino acid transport and metabolism), G (carbohydrate transport and metabolism), K (transcription), R (general function prediction only), and S (function unknown) were higher, composing approximately 50% of total functional abundance. The relative abundance of the S function was the highest, indicating that unknown functions still comprised most of the bacterial community activity in the mixed soil. Compared with the CK treatment, the functional abundances of C, E, G, K, R and S all increased significantly with the P2 treatment (10.51%, 6.64%, 8.49%, 15.30%, 8.03% and 1.71%, respectively). The functional diversity in the P1 and P3 treatments increased, while the S function decreased by 6.45% and 0.31%, respectively, compared with the CK treatment.

## Discussion

### Compound soil properties

This study found no significant differences in soil pH across the various treatments; all were alkaline, indicating that soft rock had little effect on the pH of sandy soil. In contrast, the soil nutrient content was significantly different between different treatments. The SOC, TN, and NH_4_^+^-N were highest in the P1 treatment; there was no significant difference between P1 and P2, which may be due to the point of contact between the soft rock particles and the sandy soil particles (Han, Xie & Zhang, 2012). AP, AK, and NO_3_^−^-N were highest in the P3 treatment, possibly because as the proportion of soft rock increases, the soil structure becomes compact and cohesive, meaning available nutrients were absorbed and retained in large quantities. Previous studies have suggested that the main nutrient sources of soil bacteria are root exudates and litters; the quality and quantity of nutrients provided by roots and litters for microorganisms differed in study, resulting in different soil bacterial community compositions developing under different treatments (Dai, Wang & Fu, 2017). The results of this study showed that soil physical and chemical properties had different effects on bacterial community composition, with NO_3_^−^-N, NH_4_^+^-N, and SOC having the largest overall impact. Because the soil bacterial community can also choose a specific environment to colonize, this indicated that there were synergistic changes between soil properties and the bacterial community in the process of improving sandy land with soft rock addition.

### Bacterial community structure of compound soil

Steven et al. (2013) showed that *Chloroflexi* in studied soil was highly abundant, likely due to its role in the surface biological protection layer. Rao & Burns (1990) pointed out that surface protection measures significantly affected the biological and physical properties of the top soil in their study. The results of this study showed that the dominant bacterial community differed from the conclusion of Steven et al. (2013). The relative abundance of *Actinobacteriota*, *Proteobacteria*, and *Chloroflexi* in the surface layer of compound soil were higher, and *Cloacimonadota* appeared in the P2 treatment. With refinement of the classification level, more endemic genera were found in the soil. The reason for this difference may be due to the difference in the nutrient content of the compound soil, or in the higher adaptability of endemic species to the new micro-environmental conditions caused by the addition of soft rock (Yu et al., 2020). It is also possible that the abundance of unknown organisms occurring in P2 was higher, thus mapping to more species that have not yet been classified. Some studies on the interaction between potato growth characteristics and agronomic measures have also shown that with changes in sandy soil environments, the 1:5 compound soil had a more significant effect on the increase of organic matter content and potato yield (Zhang, Wang & Sun, 2021). Moreover, the 1:5 compound soil is loosely compacted and has good air permeability, which is conducive to the expansion and growth of potato tubers (Han, Xie & Zhang, 2012).

### Species diversity of compound soils

Analysis of soil bacteria α and β diversity showed that the addition of different proportions of soft rock changed the richness and diversity indices of the bacteria; this indicated that soft rock addition improved the biological characteristics of sandy soil. The results here were similar to the bacterial community structure in the Gurbantungut Desert (Liu et al., 2019). This was because differences in soil nutrient content, pH, and other environmental factors affect the distribution of soil microbial communities, and the soils from the two areas are similar in these regards (Tiemann & Billings, 2012; Dai et al., 2017). The unknown organism fractions of P1 and P3 treatments decreased, indicating a further increase in species diversity after the addition of soft rock. Among the four treatments, the community structure of the P1 and P3 treatments was relatively similar; the α diversity of bacteria changed, and the abundance of the common species *Myxococcota* in the P1 and P3 treatments was higher than that of the P2 treatment. *Myxococcota* is a special species known to use live microbial cells or other biological macromolecules as food; it can also respond to external nutritional thresholds to regulate the differentiation of vegetative cells into stress-resistant myxospores, giving the *Myxococcota* group good adaptability to soil conditions (Li et al., 2019).

### Relationship between soil properties and bacterial communities

Analyzing differences between groups, it can be concluded that *norank_f__Roseiflexaceae* and *Gaiella* were significantly different in proportion in different treatments; both belong to the *Actinobacteria* phylum. The *Actinobacteria* phylum was mainly positively correlated with the content of NO_3_^−^-N, SOC, and NH_4_^+^-N in the compound soil. It can be seen that the *Actinobacteria* phylum was the first dominant group in the compound soil, and had relatively high relative abundance in each compound soil; this was expected, as *Actinobacteria* are known to thrive in neutral and alkaline pH soils (Sun et al., 2020). *Actinobacteria* also has a strong adhesion ability, and can become a source of bacterial storage. The mucus secreted by *Actinobacteria* can bond sandy soil, and its filamentous cells are also conducive to increasing the stability of soil, providing a certain degree of sand fixation (Mummey et al., 2006). In order to further understand the impact of changes in soil physical and chemical properties on the composition of soil bacterial communities during sandy land improvement, redundancy analysis was performed. The results showed that soil NO_3_^−^-N, NH_4_^+^-N, and SOC content had the greatest impact on the bacterial community. This result also confirmed that in the process of sandy land improvement, soil properties and microbial communities change synergistically.

## Conclusions

In a method for sandy land improvement, the addition of different proportions of soft rock changed soil physical and chemical properties and improved soil fertility. The soil bacterial community structure was also changed significantly. The dominant bacteria were *Actinobacteriota*, *Proteobacteria*, and *Chloroflexi*. The richness, diversity index, and gene copy number of soil bacteria were the highest in P1 and P3 compound soil mixtures, and the community structure between these two soils was relatively similar. Soil factors were the main factors driving the distribution of soil bacterial communities. NO_3_^−^-N, NH_4_^+^-N, and SOC were the primary factors causing differentiation of bacterial community structure, and were highly correlated with *Actinobacteria* abundance. Overall, the P1 and P3 compound soils both showed strong carbohydrate transport and metabolism capabilities. This study has good promotion prospects and feasibility in sandy land remediation, and is of great significance for the supplementation of arable land and the improvement of sandy land productivity. Under the background of energy conservation and emission reduction, it also has important reference significance for the improvement of carbon sequestration capacity of sandy land.

## Supplemental Information

10.7717/peerj.13561/supp-1 Supplemental Information 1 Raw data on soil physicochemical properties and gene copy number.

10.7717/peerj.13561/supp-2 Supplemental Information 2 Bacterial sequence dataset when the ratio of soft rock to sand is 0:1.

10.7717/peerj.13561/supp-3 Supplemental Information 3 Bacterial sequence dataset when the ratio of soft rock to sand is 1:5.

10.7717/peerj.13561/supp-4 Supplemental Information 4 Bacterial sequence dataset when the ratio of soft rock to sand is 1:2.

10.7717/peerj.13561/supp-5 Supplemental Information 5 Bacterial sequence dataset when the ratio of soft rock to sand is 1:1.

## Additional Information and Declarations

### Data Availability

The following information was supplied regarding data availability: The sequencing data is available at NCBI (SRA): SRR17498847, SRR17498846, SRR17498837, SRR17498836, SRR17498835, SRR17498834, SRR17498833, SRR17498832 and SRR17498831. BioProject: PRJNA795587; BioSample: SAMN24736255, SAMN24736256, SAMN24736257, SAMN24736258, SAMN24736259, SAMN24736260, SAMN24736261, SAMN24736262, SAMN24736263.
